# PARP and PARG inhibitors in cancer treatment

**DOI:** 10.1101/gad.334516.119

**Published:** 2020-03-01

**Authors:** Dea Slade

**Affiliations:** Department of Biochemistry, Max Perutz Labs, Vienna Biocenter (VBC), University of Vienna, 1030 Vienna, Austria

**Keywords:** poly(ADP-ribose) polymerases, poly(ADP-ribose) glycohydrolase, PARP inhibitor, PARG inhibitor, cancer therapy

## Abstract

Oxidative and replication stress underlie genomic instability of cancer cells. Amplifying genomic instability through radiotherapy and chemotherapy has been a powerful but nonselective means of killing cancer cells. Precision medicine has revolutionized cancer therapy by putting forth the concept of selective targeting of cancer cells. Poly(ADP-ribose) polymerase (PARP) inhibitors represent a successful example of precision medicine as the first drugs targeting DNA damage response to have entered the clinic. PARP inhibitors act through synthetic lethality with mutations in DNA repair genes and were approved for the treatment of *BRCA* mutated ovarian and breast cancer. PARP inhibitors destabilize replication forks through PARP DNA entrapment and induce cell death through replication stress-induced mitotic catastrophe. Inhibitors of poly(ADP-ribose) glycohydrolase (PARG) exploit and exacerbate replication deficiencies of cancer cells and may complement PARP inhibitors in targeting a broad range of cancer types with different sources of genomic instability. Here I provide an overview of the molecular mechanisms and cellular consequences of PARP and PARG inhibition. I highlight clinical performance of four PARP inhibitors used in cancer therapy (olaparib, rucaparib, niraparib, and talazoparib) and discuss the predictive biomarkers of inhibitor sensitivity, mechanisms of resistance as well as the means of overcoming them through combination therapy.

## Abstract (precis)

In this review, Slade provides an overview of the molecular mechanisms and cellular consequences of PARP and PARG inhibition. The author also highlights the clinical performance of four PARP inhibitors used in cancer therapy (olaparib, rucaparib, niraparib, and talazoparib) and discusses the predictive biomarkers of inhibitor sensitivity and mechanisms of resistance as well as the means of overcoming them through combination therapy.

Cancer is one of the most devastating diseases of our time. Uncontrolled and abnormal growth of cancer cells relies on a panel of acquired functions referred to as cancer hallmarks: sustaining proliferative signaling, enabling replicative immortality, evading growth suppressors, resisting cell death, inducing angiogenesis, activating invasion and metastasis, reprogramming energy metabolism, and evading immune destruction (Hanahan and Weinberg 2011). Acquisition of these cancer traits is facilitated by two “enabling characteristics”: genomic instability and inflammation (Hanahan and Weinberg 2011). The common denominator of genomic instability and inflammation is oxidative stress. Cancer cells experience high levels of oxidative stress. Oncogenes such as MYC and RAS induce the production of reactive oxygen species (ROS) and replication stress (Vafa et al. 2002; Maya-Mendoza et al. 2015). Inflammatory cells such as macrophages and neutrophils can induce oxidative stress themselves by releasing ROS (Grivennikov et al. 2010; Forrester et al. 2018). ROS induce DNA damage and mutations, resulting in genomic instability (Tubbs and Nussenzweig 2017). ROS also activate proinflammatory transcription factors that induce expression of inflammatory molecules (Grivennikov et al. 2010; Forrester et al. 2018). Anticancer drugs have been designed to target the whole panel of cancer traits. Arguably, targeting genomic instability and inflammation and amplifying these “enabling characteristics” to turn them into “disabling factors” is a promising way to eradicate cancer.

Over the past decade poly(ADP-ribose) polymerases (PARPs) have emerged as a new target in cancer therapy (Mateo et al. 2019). PARP inhibitors capitalize on genomic instability caused by oxidative and replication stress, as well as deficiencies in DNA repair pathways. Four PARP inhibitors, olaparib, rucaparib, niraparib, and talazoparib, have been approved by the U.S. Food and Drug Administration (FDA) and by the European Medicines Agency (EMA). In 2014, olaparib was approved as maintenance therapy for platinum-sensitive advanced ovarian cancer with germline mutations in DNA repair genes *BRCA1/2* that are required for the homologous recombination (HR) pathway of double-strand break (DSB) repair. In 2016, rucaparib was approved for advanced ovarian cancer with both germline and somatic *BRCA1/2* mutations. In 2017 and 2018, olaparib, rucaparib, and niraparib were approved for the maintenance treatment of recurrent, epithelial ovarian, fallopian tube, or primary peritoneal cancer irrespective of the *BRCA* status. Last, in 2018, olaparib and talazoparib were approved for *human epidermal growth factor receptor type 2* (HER2)-negative locally advanced or metastatic breast cancer with germline *BRCA1/2* mutations. Multiple clinical trials carried out since 2009 have demonstrated PARP inhibitor efficacy in *BRCA* mutated ovarian and breast cancer, but also prostate, pancreatic cancer, and small cell lung carcinoma (SCLC), irrespective of the *BRCA* status (Weaver and Yang 2013; Sonnenblick et al. 2015; Mirza et al. 2018; Franzese et al. 2019; Keung et al. 2019; Mateo et al. 2019; Pant et al. 2019; Pilie et al. 2019a). Inhibitors of poly(ADP-ribose) glycohydrolase (PARG) joined the stage once structures of the PARG catalytic site became available (Slade et al. 2011; Dunstan et al. 2012; Kim et al. 2012; Barkauskaite et al. 2013). Rather than synergizing with deficiencies in DNA repair pathways, PARG inhibitors seem to exploit deficiencies in replication machinery and higher levels of replication stress in cancer cells (Pillay et al. 2019).

In general, cancers with high levels of replication stress and genomic instability due to DNA repair deficiency and/or oncogene-induced increase in replication origin firing are particularly responsive to PARP and PARG inhibition. PARP and PARG inhibitors exploit and exacerbate these tumor vulnerabilities by inducing further DNA damage, preventing DNA repair and amassing unresolved replication intermediates that instigate replication and mitotic catastrophe.

## Molecular mechanisms of PARP and PARG inhibitors

**Figure 1.**
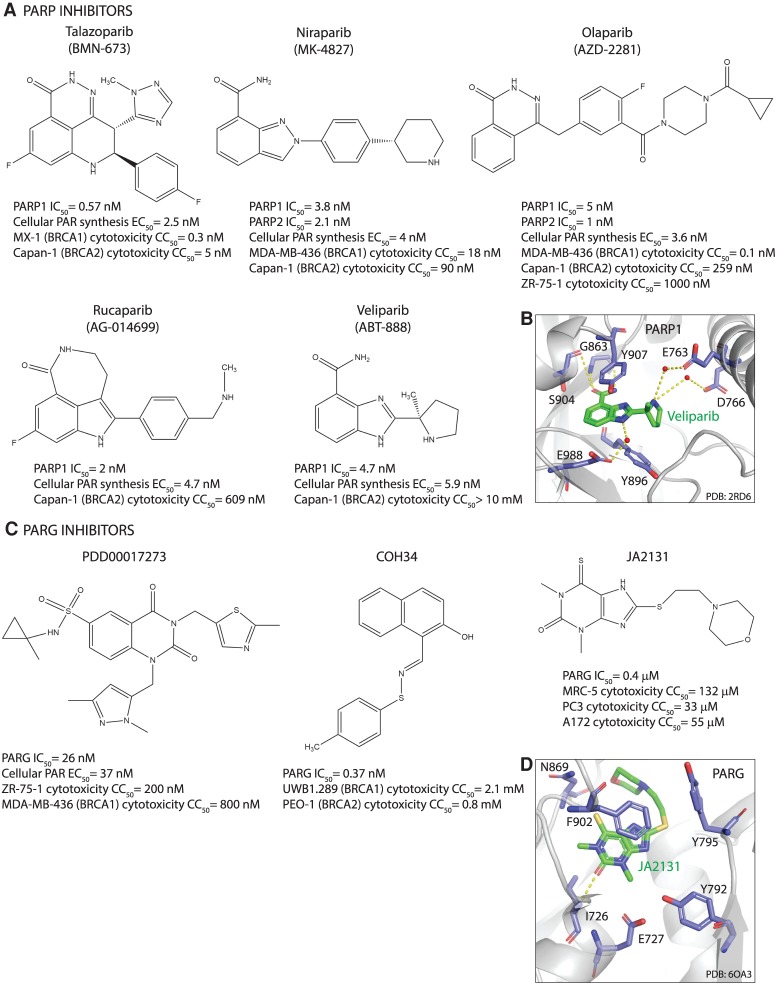
Structures of PARP and PARG inhibitors. (*A*,*C*) Chemical structures. IC_50_ denotes half-maximal inhibitory concentration based on measurements of PARP/PARG activity in vitro. Cellular PAR synthesis EC_50_ denotes half-maximal effective concentration determined by measuring PAR levels in cellular extracts treated with inhibitors. Cytotoxicity CC_50_ denotes half-maximal cytotoxic concentration determined by measuring cell viability after PARP/PARG inhibitor treatment. (*B*,*D*) X-ray structures. (*B*) Veliparib bound to PARP1 active site (PDB: 2RD6). (*D*) JA2131 bound to PARG active site (PDB: 6OA3). Inhibitors are labeled in green, PARP1/PARG residues in the binding pocket are labeled in blue, water molecules are shown as red dots, and hydrogen bonds are represented by yellow dashes.

PARPs synthesize poly(ADP-ribose) (PAR) from NAD, releasing nicotinamide as the reaction product (Okayama et al. 1977). PARP1, as the major producer of cellular PAR, is activated by binding DNA lesions (Benjamin and Gill 1980a,b). Catalytic activation of PARP1 is a multistep process of binding to DNA through N-terminal zinc fingers (ZnF), unfolding of the helical domain (HD), binding of NAD to the catalytic pocket, and PAR catalysis (Langelier et al. 2012; Eustermann et al. 2015). The first PARP1 inhibitor was nicotinamide itself (Clark et al. 1971), followed by 3-aminobenzamide (3-AB) (Purnell and Whish 1980). All subsequently developed PARP1 inhibitors contain nicotinamide/benzamide pharmacophores and compete with NAD for the catalytic pocket of PARPs (Fig. 1; Ferraris 2010; Steffen et al. 2013). PARP1 inhibitors dock into the catalytic site by forming hydrogen bonds with Gly, Ser, and Glu as well as hydrophobic stacking interactions with two Tyr residues within the nicotinamide-binding pocket (Fig. 1; Ferraris 2010). Given the high degree of conservation of the catalytic pocket among different PARPs, additional interactions are required for selective inhibition (Steffen et al. 2013). A screen for more potent and selective inhibitors identified different scaffolds from which new-generation PARP1 inhibitors evolved; phthalazinone and tetrahydropyridophthalazinone served as a scaffold for olaparib and talazoparib, benzimidazole and indazole carboxamide for veliparib and niraparib, tricyclicindole lactam for rucaparib (Banasik et al. 1992; White et al. 2000; Canan Koch et al. 2002). Olaparib was the first PARP inhibitor that entered clinical trials due to its selectivity for inhibiting PARP1/2 as well as its potency, oral availability, and favorable pharmacokinetic and pharmacodynamic properties (Menear et al. 2008; Fong et al. 2009). All clinically relevant PARP1/2 inhibitors have high catalytic activity with IC_50_ in the low nanomolar range and inhibit PARP1 and PARP2 with similar efficiency (Fig. 1; Menear et al. 2008; Jones et al. 2009; Shen et al. 2013, 2015; Wang et al. 2016a).

Despite improved selectivity, many PARP1/2 inhibitors are not highly selective over other family members (Wahlberg et al. 2012; Papeo et al. 2014; Thorsell et al. 2017). Among clinically relevant inhibitors, veliparib is the most selective PARP1/2 inhibitor, followed by niraparib (Thorsell et al. 2017). Their selectivity is based on formation of a PARP1/2-unique water-mediated hydrogen bond interaction with a regulatory subdomain residue (D766 in PARP1), which is conserved in PARP1/2 but not in other PARPs (Fig. 1). Compared with veliparib, which exhibits >100-fold higher selectivity for PARP1/2 compared with other family members, olaparib and talazoparib show only 15-fold to 20-fold higher selectivity (Thorsell et al. 2017). Rucaparib is the least selective clinical PARP1 inhibitor, which inhibits different PARPs (PARP1, PARP2, PARP5A, and PARP5B) as well as mono(ADP-ribosyl) transferases PARP3, PARP4, PARP10, PARP15, and PARP16 (Thomas et al. 2007; Wahlberg et al. 2012). Moreover, some PARP inhibitors such as rucaparib and niraparib also inhibit non-PARP targets, albeit with lower efficiency; rucaparib inhibits hexose-6-phosphate dehydrogenase (H6PD), while niraparib inhibits deoxycytidine kinase (DCK) (Knezevic et al. 2016). Such cross-inhibition may potentiate cancer cell death, as in the case of rucaparib and PARP/H6PD inhibition, but may also be detrimental for combination therapy with niraparib and nucleoside analogs such as gemcitabine due to cross-inhibition of DCK required for their activation (Knezevic et al. 2016).

**Figure 2.**
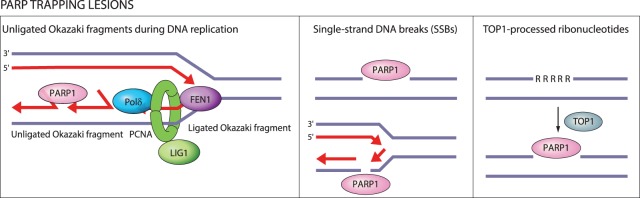
DNA lesions recognized by PARP1 as potential PARP-trapping sites. Unligated Okazaki fragments are DNA replication intermediates. Single-strand DNA breaks (SSBs) are a frequent form of endogenous DNA damage and are particularly hazardous for replication forks. Ribonucleotides incorporated into DNA need to be removed by RNase H2-mediated ribonucleotide excision repair. In RNase H2-deficient cells these ribonucleotides are removed by topoisomerase I (TOP1)-mediated excision. TOP1 cleavage results in nicks, covalent TOP1–DNA adducts, and single-strand DNA gaps that can engage PARP1.

In addition to inhibiting PARP catalytic activity, PARP inhibitors also trap PARP1 and PARP2 on DNA (Murai et al. 2012, 2014a). PARP1 is the dominant target for DNA trapping by PARP inhibitors, as depletion of PARP1—but not PARP2—reduces sensitivity to PARP inhibitors (Murai et al. 2012). PARP1 being the relevant target is consonant with its high nuclear abundance and its requirement for synthetic lethality with HR deficiency (Amé et al. 1999; Ronson et al. 2018; Murai and Pommier 2019). Entrapment of PARP1 on DNA can be determined based on the shift in distribution of PARP1 from nuclear-soluble to chromatin-bound fraction (Murai et al. 2012). PARP entrapment can occur on DNA-strand breaks as well as topoisomerase I (TOP1)-processed ribonucleotides and unligated Okazaki-fragment intermediates of DNA replication (Fig. 2; Strom et al. 2011; Hanzlikova et al. 2018; Zimmermann et al. 2018). Once trapped, PARP1 cannot dissociate from DNA due to inhibition of its catalytic activity, which is required for repulsion between auto-PARylated PARP1 and DNA (Pommier et al. 2016). Catalytic inhibition of PARP1 auto-PARylation is thus a prerequisite for PARP1-DNA trapping (Hopkins et al. 2015).

The catalytic inhibitory effects of the clinically relevant PARP inhibitors olaparib, rucaparib, niraparib, and talazoparib are comparable; however, their potency in trapping PARP–DNA complexes varies considerably, which is why PARP1 trapping was proposed to rely on allosteric changes in the PARP1 DNA-binding domain induced by the PARP inhibitor binding to the D-loop at the outer border of the NAD site (Murai et al. 2012, 2014a). Talazoparib exhibits the highest trapping efficiency (talazoparib >> niraparib > olaparib = rucaparib >> veliparib) and has the most rigid structure (Murai et al. 2014a). Veliparib is one of the weakest PARP1/2 inhibitors with low PARP trapping efficiency (Murai et al. 2012).

PARG hydrolyzes ribose–ribose bonds within PAR with high specific activity and processivity, particularly after DNA damage (Wielckens et al. 1982; Hatakeyama et al. 1986; Alvarez-Gonzalez and Althaus 1989). PARG has a macro domain that binds ADP-ribose moiety and a PARG-specific loop with conserved glutamates that cleave ribose–ribose bonds in an exoglycohydrolase mode (Slade et al. 2011; Dunstan et al. 2012; Kim et al. 2012; Barkauskaite et al. 2013). The first PARG inhibitors gallotannin and GPI-16552 showed low activity in vitro and off-target effects in cells (Falsig et al. 2004; Erdèlyi et al. 2005). ADP-HPD and rhodanine-based PARG inhibitors (RBPIs) are potent and specific inhibitors, but lack cell permeability (Slama et al. 1995; Finch et al. 2012). The quinazolinedione-type PARG inhibitor PDD00017273 inhibits PARG selectively and with high efficiency, is cell-permeable and cell-active, but has limited bioavailability, which makes it unsuitable for clinical application (James et al. 2016). The naphthalen-type PARG inhibitor COH34 is a potent, specific, and cell-permeable inhibitor with a terminal half-life of 3.9 h, and may thus prove a good candidate for clinical studies (Chen and Yu 2019). Chemical library screening identified thioxanthine/methylxanthine derivatives JA2–4 and JA2131 as potent, specific, cell-permeable, and cell-active PARG inhibitors, which are also likely to show good bioavailability given their structural similarity with caffeine (Houl et al. 2019). PARG inhibitors compete with PAR for the PARG active site by occupying the subsite normally occupied by the adenine moiety of ADP-ribose (Fig. 1; James et al. 2016; Chen and Yu 2019; Houl et al. 2019).

## Cellular mechanisms of PARP and PARG inhibitors

### Functions of PARP1 and PARG in DNA repair and replication fork protection

Nuclear functions of PARP1 and PARG in DNA repair, replication fork protection, and transcription regulation are critical for understanding the mechanism of action of PARP inhibitors. PARP1 is involved in different pathways of DNA repair, including single-strand DNA break (SSB) repair, nucleotide excision repair (NER), alternative nonhomologous end-joining (alt-NHEJ), and homologous recombination (HR) (Ray Chaudhuri and Nussenzweig 2017). PARP1 and PARG are also critical for preserving the integrity of replication forks under conditions that induce replication stress (Hanzlikova and Caldecott 2019). PARP1 or PARG depletion or inhibition exert the most profound effects on SSB repair and replication fork stability.

**Figure 3.**
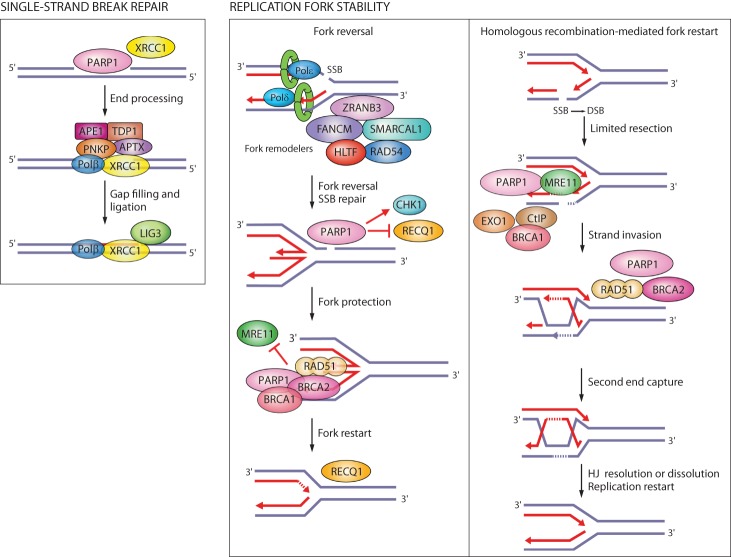
Single-strand break (SSB) repair and replication fork protection by PARP1. PARP1 acts as a sensor of SSBs and recruits XRCC1. XRCC1 is a scaffold for the recruitment of proteins that process damaged termini, DNA polymerase β that fills the gap, and DNA ligase III that seals the nick. PARP1 rescues damaged replication forks through fork reversal or homologous recombination (HR). SSBs on the leading strand trigger fork reversal by fork remodeling proteins. PARP1 promotes fork reversal by inhibiting the RECQ1 helicase involved in fork restart. PARP1 stabilizes RAD51 filaments on reversed forks and together with BRCA1 and BRCA2 protects forks from degradation by the MRE11 nuclease. If forks collapse when encountering an SSB on the lagging strand, PARP1 promotes HR-mediated fork repair and restart by recruiting MRE11, EXO1, and BRCA1-CtIP for end resection, and BRCA2 for RAD51 filament formation.

In SSB repair, PARP1 activity is important for the recruitment of the scaffold protein XRCC1 to the sites of DNA damage, while PARG regulates XRCC1 dissociation (Fig. 3; El-Khamisy et al. 2003; Okano et al. 2003; Fisher et al. 2007; Chen and Yu 2019). PARP1/XRCC1-dependent SSB repair was implicated as an alternative pathway of Okazaki fragment processing (Fig. 2; Hanzlikova et al. 2018).

PARP1 interacts with DNA replication machinery and is active during S phase and in response to replication stress (Jump et al. 1979; Anachkova et al. 1989; Dantzer et al. 1998; Simbulan-Rosenthal et al. 1998; Bryant et al. 2009). Replication stress leads to uncoupling between DNA polymerase and helicase activities, which generates single-stranded DNA (ssDNA). RPA binds ssDNA and recruits the S/G2 checkpoint kinase ATR. ATR suppresses silent origin firing and activates the checkpoint kinase CHK1 to induce cell cycle arrest (Zeman and Cimprich 2014). By preventing unscheduled origin firing, replication checkpoints prevent accumulation of ssDNA and exhaustion of RPA, and thereby safeguard against fork breakage (Toledo et al. 2013). Replication stress generates SSBs and exposes unligated Okazaki fragments as DNA substrates for PARP1 binding (Hanzlikova and Caldecott 2019). In response to replication stress, PARP1 slows down replication forks to promote fork reversal by antagonizing the RECQ1 helicase (Yang et al. 2004; Sugimura et al. 2008; Bryant et al. 2009; Ray Chaudhuri et al. 2012; Berti et al. 2013), protects replication forks from degradation by the MRE11 nuclease (Ying et al. 2012), stabilizes RAD51 nucleofilaments at stalled forks together with PARP2 (Ronson et al. 2018), and activates the S-phase checkpoint kinase CHK1 (Fig. 3; Min et al. 2013). PARG localizes at replication forks by binding PCNA and promotes recovery from prolonged replication stress (Mortusewicz et al. 2011; Illuzzi et al. 2014; Kaufmann et al. 2017). PARP1 also regulates replication and DNA repair at the transcription level by stimulating activity of the transcription factor E2F1, which regulates the expression of replication and HR genes (Simbulan-Rosenthal et al. 1998, 2003).

Replication forks are prone to breakage if they encounter an SSB, which is why homologous recombination is a critical pathway for repairing replication forks to prevent fork collapse (Ait Saada et al. 2018). PARP1 contributes to the homologous recombination pathway of DSB repair by promoting rapid recruitment of MRE11, EXO1, BRCA1, and BRCA2 to DNA damage sites (Fig. 3; Haince et al. 2008; Li and Yu 2013; Zhang et al. 2015a,b). The MRE11 nuclease is responsible for the early processing of DNA lesions, while EXO1 and BRCA1-CtIP contribute to extensive end resection. BRCA2 is required for the loading of RAD51 filaments onto ssDNA generated by end resection. PARP1 also counteracts nonhomologous end-joining (NHEJ) as the alternative pathway of DSB repair by preventing the binding of the NHEJ protein Ku to DNA ends (Hochegger et al. 2006; Wang et al. 2006; Patel et al. 2011; Yang et al. 2018).

In addition to PARP1 and PARG, other members of the PARP family have also been implicated in DSB repair and replication fork stability, most notably PARP2, PARP3, PARP10, and PARP14 (Martin-Hernandez et al. 2017), and may contribute to cellular phenotypes of PARP1/2 inhibitors given that some of them exhibit weaker target specificity.

### Synthetic lethality between PARP or PARG inhibitors and genomic instability in cancer cells

Genomic instability underlies the ability of cancer cells to acquire different tumorigenic properties. Genomic instability entails chemical alterations in DNA known as mutations as well as changes in the chromosome number or structure defined as chromosomal instability. Genomic instability arises due to high levels of DNA damage caused by oxidative or replication stress, defects in DNA repair pathways, and/or dysfunctional surveillance mechanisms that fail to trigger cellular senescence or apoptosis (Tubbs and Nussenzweig 2017). Cancer cells experience high levels of oxidative and replication stress, resulting in high mutational rates (Bartkova et al. 2005; Gorgoulis et al. 2005; Dobbelstein and Sørensen 2015; Macheret and Halazonetis 2015; Kotsantis et al. 2018). Activation of oncogenes such as MYC, RAS, and cyclin E1 (CCNE1) induces replication stress by promoting premature entry into S phase, increasing replication origin firing, changing replication fork rates, causing nucleotide depletion, and inducing replication–transcription conflicts (Bester et al. 2011; Kotsantis et al. 2016; Macheret and Halazonetis 2018). Replication stress leads to accumulation of replication errors and DNA lesions that compromise fork stability and require DNA repair pathways to restore fork progression (Dobbelstein and Sørensen 2015; Macheret and Halazonetis 2015; Kotsantis et al. 2018). Many cancers have germline or somatic mutations in DNA repair genes. Mutations in tumor suppressor genes such as the cell cycle checkpoint gene *TP53* are common across different cancer types and allow cancer cells to escape senescence or apoptosis and continue proliferating in the presence of DNA damage (The Cancer Genome Atlas Research Network 2011; Kandoth et al. 2013; Nik-Zainal et al. 2016; Robinson et al. 2017; Hafner et al. 2019).

Genotoxic agents have been used routinely in cancer therapy in order to induce high levels of DNA damage that render cancer cells particularly vulnerable due to their high proliferation rates. These include ionizing radiation and chemotherapeutic drugs that damage DNA by inducing DSBs (e.g., bleomycin, doxorubicin, topoisomerase inhibitors), intrastrand or interstrand DNA cross-links (platinum compounds; e.g., cisplatin, carboplatin, and oxaliplatin), DNA base alkylation (e.g., temozolomide), or that interfere with DNA replication such as nucleoside and base analogs (e.g., gemcitabine and 5-fluorouracil). Mitotic drugs that inhibit cell division such as taxanes (e.g., docetaxel and paclitaxel) are also used in chemotherapy. Chemotherapy is often combined with radiotherapy. Synergistic effects are additionally achieved in patients with genetic deficiencies in DNA repair pathways. For example, platinum drugs (carboplatin) improved response rate in *BRCA* mutated advanced triple-negative breast cancer (TNBC) patients and are more effective than taxanes (Telli et al. 2016; Tutt et al. 2018). However, genotoxic agents also affect normal cells and have severe side effects such as myelosuppression.

**Figure 4.**
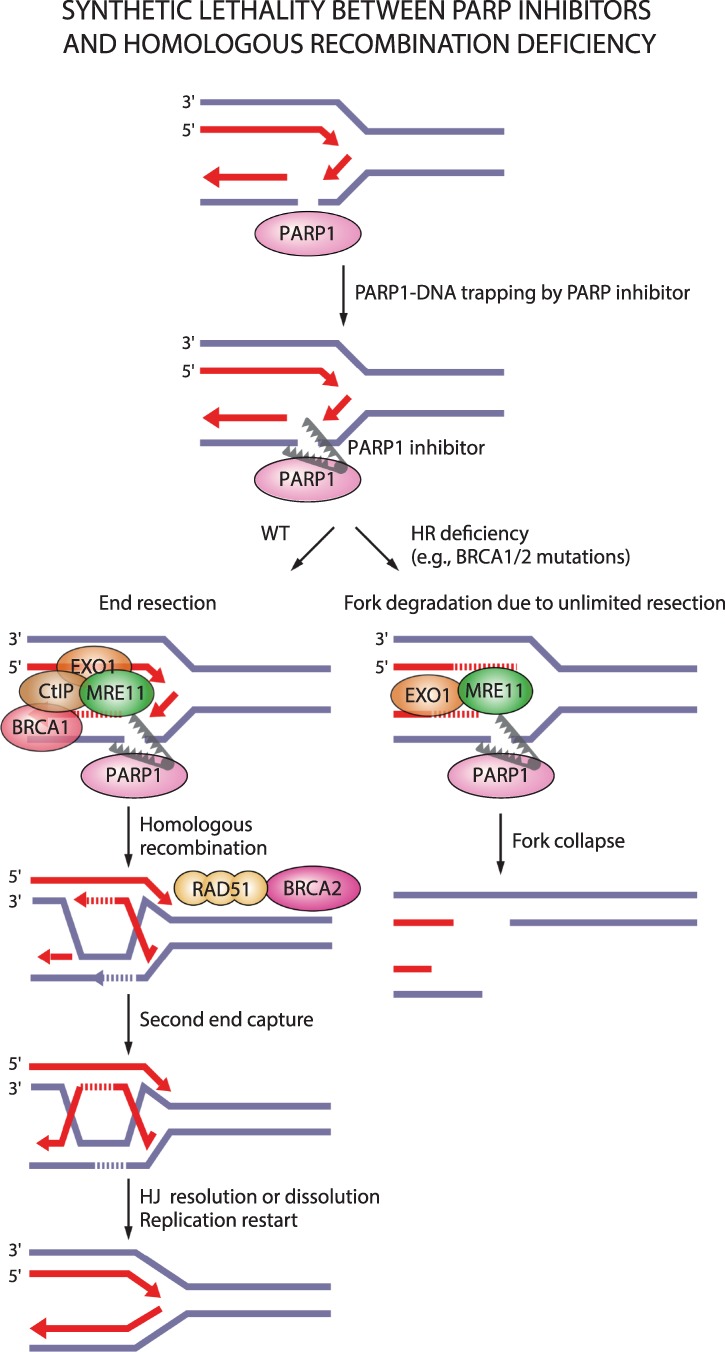
Synthetic lethality between PARP inhibitors and homologous recombination deficiency. PARP entrapment on DNA lesions blocks replication machinery and loss of PARP activity prevents fork protection, fork reversal, and fork restart. This results in DSBs that need to be repaired by homologous recombination. In the case of homologous recombination deficiency due to, for example, mutations in *BRCA1/2*, PARP1-trapping lesions elicit excessive fork degradation by the MRE11 nuclease, the activity of which is unrestrained in the absence of BRCA1/2 and PARP1. This results in fork collapse.

Precision medicine has revolutionized cancer therapy by putting forth the concept of selective targeting of cancer cells. PARP inhibitors represent a successful example of precision medicine applied in the clinic. PARP inhibitors act through synthetic lethality, whereby genetic DNA repair defects are enhanced by drug-induced defects in a compensatory pathway (Lord and Ashworth 2017). Two seminal studies showed how PARP inhibitors specifically kill HR-deficient cells mutated in *BRCA1/2* (Fig. 4; Bryant et al. 2005; Farmer et al. 2005). Carriers of heterozygous *BRCA1/2* mutations are sensitive to PARP inhibitor treatment as they lose the wild-type allele during tumorigenesis and thereby become BRCA1/2-null. Since this first example of synthetic lethality between genetic defects and PARP inhibitors, it has become clear that oxidative stress and genomic instability, manifested not just through mutations in DNA repair proteins but also replication stress, sensitize cells to PARP and PARG inhibitors (Bryant et al. 2005; Farmer et al. 2005; McCabe et al. 2006; Bunting et al. 2010; Lord and Ashworth 2012, 2017; McLellan et al. 2012; Murai et al. 2012; Dréan et al. 2016; Gravells et al. 2017; Zimmermann et al. 2018; Chen and Yu 2019; Giovannini et al. 2019; Pillay et al. 2019). In addition to *BRCA1/2*, mutations in DNA damage response genes such as *ATM*, *PRKDC*, *ATR*, *RPA1*, *DSS1*, *NBN*, *RAD51*, *RAD54*, *CHEK1*, *CHEK2*, *FANC* genes, *ERCC1*, *POLB*, *FEN1*, and *CDK12* have shown synthetic lethality in combination with PARP inhibitors (Bryant and Helleday 2006; McCabe et al. 2006; Murai et al. 2012; Postel-Vinay et al. 2013; Bajrami et al. 2014). Synthetic lethality between mutations in HR-related genes and PARP inhibition was confirmed by CRISPR screens, which enable high-throughput investigation of synthetic lethal interactions (Zimmermann et al. 2018).

**Figure 5.**
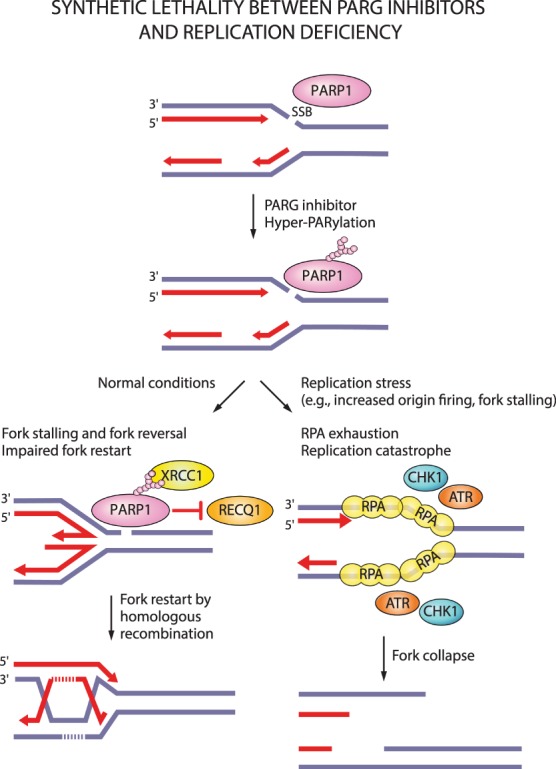
Synthetic lethality between PARG inhibitors and replication stress. PARG inhibition increases PARylation levels and may prevent dissociation of PARP1 and PAR-binding repair proteins (e.g., XRCC1) from DNA damage sites. Loss of PARG activity causes fork stalling and impairs restart of reversed forks. Forks can presumably restart by homologous recombination. Under replication stress conditions, increased origin firing and prolonged fork stalling generates excessive ssDNA and causes RPA exhaustion. Such replication catastrophe results in fork collapse.

In contrast to PARP inhibitors, a clear correlation between HR deficiency and synthetic lethality with PARG inhibitors is lacking. Depletion of the HR proteins BRCA1/2, PALB2, ABRAXAS, and BARD1 in MCF7 breast cancer cells was shown to elicit synthetic lethal interactions with PARG depletion or PARG inhibition (with gallotannin or PDD00017273) (Fathers et al. 2012; Gravells et al. 2017). The PARG inhibitor COH34 efficiently kills *BRCA* mutated or olaparib-resistant ovarian and breast cancer cells (Chen and Yu 2019). However, PARG depletion did not show synthetic lethality with *BRCA1* mutations in different cancer cell lines (Noll et al. 2016), the PARG inhibitor JA2131 efficiently killed *BRCA*-proficient cancer cells (Houl et al. 2019), and only one out of six tested ovarian cancer cells with *BRCA1/2* mutations showed sensitivity to PARG inhibition with PDD00017273 (Pillay et al. 2019). Instead, synthetic lethal interactions with the PARG inhibitor PDD00017273 involve replication-associated genes such as *TIMELESS*, *HUS1*, and *RFC2* (Fig. 5; Pillay et al. 2019).

### Amplifying genomic instability with PARP and PARG inhibitors

**Figure 6.**
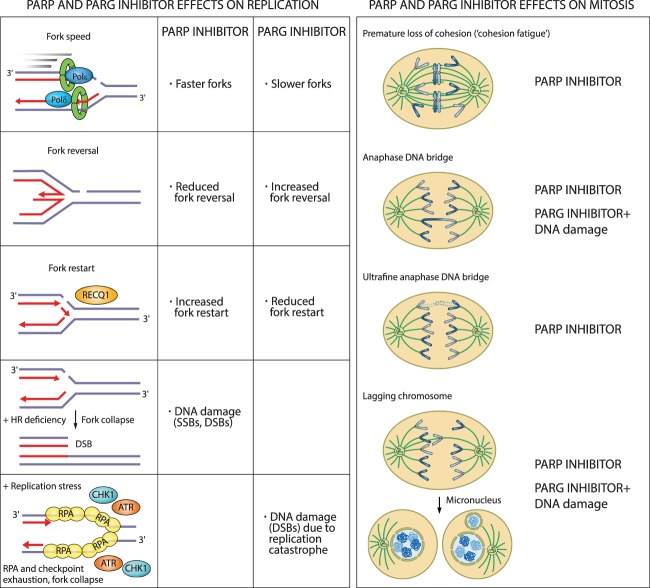
Cellular consequences of PARP and PARG inhibition on replication and mitosis. PARP inhibitors increase replication fork rate, reduce fork reversal, and cause premature fork restart. In HR-deficient cells, PARP inhibitors cause fork collapse and DSBs. PARG inhibitors reduce fork rate, increase fork reversal, and impair fork restart. In the presence of replication stress, PARG inhibitors cause replication catastrophe. Destabilization of replication forks causes mitotic defects and death by mitotic catastrophe. PARG inhibitors cause mitotic defects in combination with DNA-damaging agents.

PARP1 depletion or inhibition increases replication fork speed, impairs replication fork reversal, and causes untimely fork restart following replication stress (Fig. 6). This leads to an accumulation of DNA damage in S-phase cells, S-phase stalling, and G2 delay (Sugimura et al. 2008; Bryant et al. 2009; Ray Chaudhuri et al. 2012; Berti et al. 2013; Dale Rein et al. 2015; Farrés et al. 2015; Maya-Mendoza et al. 2018; Michelena et al. 2018; Ronson et al. 2018). In PARP1-depleted or inhibited cells, DSBs arise due to deprotection of stalled replication forks and their degradation by the MRE11 nuclease, due to impaired fork reversal, or from unligated Okazaki fragments encountered by replication forks (Lonn and Lonn 1985; Ray Chaudhuri et al. 2012; Ying et al. 2012; Hanzlikova et al. 2018).

In line with the opposing catalytic activities of PARP1 and PARG, PARG depletion or inhibition show opposite effects. PARG depletion or inhibition slows down replication forks, causes accumulation of reversed forks and ssDNA gaps, and prevents fork restart through suppression of the RECQ1 helicase (Fig. 6; Illuzzi et al. 2014; Ray Chaudhuri et al. 2015; Gravells et al. 2017; Houl et al. 2019; Pillay et al. 2019). This causes cell cycle stalling in the S/G2 phase, accumulation of ssDNA as judged by RPA foci, and accumulation of DNA damage, as indicated by pan-nuclear γH2AX staining (Illuzzi et al. 2014; Pillay et al. 2019). Prolonged exposure of PARG-depleted cells to replication stress results in the loss of RPA foci, indicating RPA exhaustion due to replication stress-induced formation of ssDNA—a concept known as replication catastrophe (Illuzzi et al. 2014; Toledo et al. 2017). PARP1 or PARG depletion or inhibition sensitize cells to DNA-damaging agents and cause increased DNA damage levels due to decreased HR efficiency (Rottenberg et al. 2008; Ame et al. 2009; Min et al. 2010; Shirai et al. 2013; Murai et al. 2014b; Dréan et al. 2016; James et al. 2016; Gravells et al. 2018; Houl et al. 2019). Collectively, loss or inhibition of PARP1 or PARG destabilize replication forks and cause fork breakage, particularly under conditions of HR deficiency, oxidative and replication stress, or exogenous DNA damage.

Impaired fork progression and accumulation of DNA damage are more pronounced in PARP inhibitor-treated cells compared with PARP-depleted cells, which suggests that PARP inhibition does more than just inhibit PARP1 catalytic activity. For example, PARP inhibition increases fork rate and reduces fork reversal following camptothecin exposure more than PARP depletion (Sugimura et al. 2008; Ray Chaudhuri et al. 2012; Maya-Mendoza et al. 2018). Furthermore, PARP inhibition results in more strand breaks (SSBs and DSBs), as judged by γH2AX foci (Murai et al. 2012) and delayed SSB repair after ionizing radiation or alkylation damage (Godon et al. 2008; Strom et al. 2011). Consequently, PARP inhibition exhibits stronger synthetic lethality with HR deficiency compared with PARP depletion (Bryant et al. 2005; Ronson et al. 2018).

In fact, PARP inhibitor cytotoxicity was found to correlate with the strength of PARP–DNA entrapment rather than a reduction in PARP1 catalytic activity (Murai et al. 2012; Pettitt et al. 2013). A combination of the chemical inhibition of PARP1 catalytic activity and the physical obstruction caused by PARP–DNA entrapment seems to be responsible for the greater cytotoxicity of PARP inhibitors compared with PARP depletion. The physical obstruction caused by PARP–DNA entrapment exacerbates replication problems caused by loss of PARP activity and induces mitotic phenotypes of premature loss of cohesion or anaphase DNA bridges, which are not observed upon PARP depletion (Murai et al. 2012; Shen et al. 2015; Kukolj et al. 2017; Schoonen et al. 2017). Therefore, the cytotoxicity of PARP inhibitors seems to arise from an accumulation of replication problems, which are carried over into mitosis resulting in death by mitotic catastrophe (Slade 2019).

PARP and PARG inhibitors activate the S/G2 checkpoint kinases ATR and CHK1 (Ray Chaudhuri et al. 2015; Colicchia et al. 2017; Kim et al. 2017; Maya-Mendoza et al. 2018; Pillay et al. 2019), which generally halt the cell cycle to allow DNA repair and completion of DNA replication before mitotic entry (Lecona and Fernandez-Capetillo 2018). In accordance with the activation of the ATR checkpoint, PARG inhibitor PDD00017273-treated cells stall in the S/G2 phase without progressing into mitosis and assume a “fried egg” morphology (Pillay et al. 2019). Conversely, despite the activation of ATR and CHK1, cells treated with PARP inhibitors progress into mitosis and exhibit different mitotic defects, which arise from problems during S phase (Fig. 6; Colicchia et al. 2017; Kim et al. 2017; Maya-Mendoza et al. 2018).

As explained above, fork destabilization caused by PARP inhibition results in DSBs. DSBs cannot be repaired by the HR pathway in BRCA-deficient cells, which forms the basis for synthetic lethality approaches with PARP inhibitors. Moreover, PARP inhibition itself induces HR deficiency by reducing the expression of the E2F1 target genes involved in DNA replication and cell cycle regulation (e.g., *PCNA*, *MCM7*, and *CCNA2*) and HR factors such as *BRCA1/2* and *RAD51*, as shown in prostate and small cell lung cancer (Byers et al. 2012; Schiewer et al. 2018). A contingency pathway for DSB repair, NHEJ, which is functional throughout the cell cycle, is thought to compensate for inactive HR in PARP inhibitor-treated cells. NHEJ is an error-prone pathway that can lead to small mutations as well as chromatid fusions (Bunting et al. 2010). Fusion of two broken sister chromatids, chromosomes, or telomeres during interphase can generate dicentric chromosomes visible as radial fusions in metaphase and as anaphase DNA bridges (Ganem and Pellman 2012). Chromatid fusions also give rise to acentric chromosomes, which cannot attach to the mitotic spindle and cannot segregate accurately during anaphase, thus appearing as lagging chromosomes. During telophase and cytokinesis, acentric or lagging chromosomes obtain their own nuclear envelope and form micronuclei (Fenech et al. 2011). Chromosomes within micronuclei are prone to chromothripsis, whereby reduced and asynchronous DNA replication results in DNA damage and chromosome fragmentation (Crasta et al. 2012). DNA released from micronuclei triggers cGAS accumulation and activation of proinflammatory response (Harding et al. 2017; Mackenzie et al. 2017). Following chromothripsis, fragmented chromosomes can assemble randomly, resulting in chromosome rearrangements (Luijten et al. 2018). Chromatid breaks, radial chromosomes, anaphase DNA bridges, lagging chromosomes, and micronuclei are all common in BRCA1/2-deficient cells treated with PARP inhibitors (Fig. 6; Bunting et al. 2010; Schoonen et al. 2017).

Genomic regions called common fragile sites (CFSs) are particularly sensitive to impaired fork progression. CFS are found within long genes and are prone to form abnormal replication intermediates due to transcription–replication conflicts (Helmrich et al. 2011). CFSs are late-replicating and remain underreplicated at the G2/M transition (Le Beau et al. 1998). Underreplicated CFSs remain connected through thin threads of DNA in mitosis known as ultrafine anaphase DNA bridges (Le Beau et al. 1998). Replication intermediates that remain unresolved during mitosis are marked by 53BP1 in G1 cells (Lukas et al. 2011). Replication stress induced by PARP inhibitors gives rise to ultrafine anaphase DNA bridges in mitotic cells and 53BP1-positive nuclear bodies in G1 cells (Gemble et al. 2015; Michelena et al. 2018). Furthermore, PARP inhibition during S phase causes weakening of sister chromatid cohesion, resulting in premature loss of cohesion (“cohesion fatigue”) and chromosome alignment problems in metaphase (Fig. 6; Kukolj et al. 2017).

It is clear that mitotic defects in PARP-inhibitor treated cells arise from destabilization of replication forks and DNA damage acquired during S phase. Replication stress-induced mitotic defects result in death by mitotic catastrophe (Dale Rein et al. 2015; Gemble et al. 2015; Majuelos-Melguizo et al. 2015; Colicchia et al. 2017; Kukolj et al. 2017; Schoonen et al. 2017; Maya-Mendoza et al. 2018; Michelena et al. 2018). Mitotic catastrophe is a special type of cell death whereby cells die by apoptosis or slip out of mitosis through multinucleation or macronucleation due to chromosome missegregation, as well as micronucleation that results from lagging or acentric chromosomes (Galluzzi et al. 2018).

While HR-deficient cancer cells were shown to respond better to PARP inhibitors, PARP inhibitors are also effective in HR-proficient cells that experience high levels of oxidative and replication stress. Indeed, anaphase DNA bridges, lagging chromosomes, micronuclei, ultrafine anaphase DNA bridges, and premature loss of cohesion occur in HR-proficient cells exposed to PARP inhibitors (Majuelos-Melguizo et al. 2015; Kukolj et al. 2017; Schoonen et al. 2017; Michelena et al. 2018). Moreover, PARP inhibitor efficacy was shown to correlate with basal levels of replication stress in cancer cells (Kukolj et al. 2017).

Unlike PARP inhibitors, the PARG inhibitor PDD00017273 exhibits cytostatic rather than cytotoxic effects by causing a replication catastrophe that is not transferred into mitosis but remains contained in interphase (Pillay et al. 2019). PARP trapping is most likely the reason why PARP inhibitors lead to mitotic catastrophe and are more potent in killing cells compared with PARG inhibitors. However, the combination of PARG inhibitors with cell cycle checkpoint inhibitors such as CHK1 or combination of PARG inhibition/depletion with DNA-damaging agents allows cells to progress into mitosis where they experience various mitotic abnormalities (Koh et al. 2004; Ame et al. 2009; Min et al. 2010; Gravells et al. 2018; Pillay et al. 2019; Slade 2019). For example, PARG-depleted cells or PARG hypomorphic cells lacking nuclear and cytoplasmic PARG isoforms show centrosome amplification, centrosome fragmentation, multipolar spindles, chromosome misalignment, and missegregation, which are more pronounced after exposure to ionizing radiation (Ame et al. 2009; Min et al. 2010). PARG inhibition coupled with ionizing radiation also yields aberrant spindle formation and metaphase arrest (Fig. 6; Gravells et al. 2018).

In sum, cytotoxicity of PARP inhibitors is a multistage process of the destabilization of replication forks through PARP entrapment and loss of PARP activity, the generation of unresolved replication intermediates and DSBs, their transmission into mitosis, and the induction of mitotic defects (premature loss of cohesion, misalignment, missegregation) that ultimately result in mitotic catastrophe. The cytostatic effects of PARG inhibitors involve fork stalling, which results in replication catastrophe and cell cycle arrest in S/G2.

### Targeting transcription, RNA metabolism, and ribosome biogenesis through PAPR inhibition

In addition to DNA repair and replication fork stability, PARP1 is also implicated in gene expression regulation, RNA processing, and ribosome biogenesis, which may contribute to cellular effects of PARP inhibition (Weaver and Yang 2013; Feng et al. 2015) and give rise to synthetic lethal interactions with transcription and splicing factors identified in CRISPR screens (Zimmermann et al. 2018). PARP1 modulates the activity of different transcription regulators implicated in cancer (e.g., p53, nuclear receptors) or inflammation (e.g., NF-κB) (Schiewer and Knudsen 2014; Bai 2015). Hence, transcriptional deregulation may sensitize cancer cells to PARP inhibitors, as shown for DNA-repair proficient HER2-positive breast cancer cells whereby NF-κB overactivation is attenuated through PARP inhibition (Nowsheen et al. 2012). PARP inhibitors are also effective in Ewing's sarcomas by blocking, on the one hand, PARP1-dependent transcriptional activation effects of ETS gene fusions such as EWS-FLI-1, and by exacerbating DNA damage on the other (Brenner et al. 2012). Furthermore, PARP inhibitors reduce rDNA transcription and ribosome biogenesis in BRCA1/2-proficient cancer cells by preventing DDX21 ADP-ribosylation, and thereby reduce breast cancer growth (Kim et al. 2019).

### Determinants of PARP inhibitor sensitivity in cancer cells

Since the first example of synthetic lethality between PARP inhibitors and *BRCA1/2* mutations, it has become clear that any form of HR deficiency in tumors that phenocopies *BRCA1/2* mutations, often referred to as BRCAness, may sensitize cells to PARP inhibitors (Lord and Ashworth 2016). Different patient biomarkers have been used to assess HR deficiency as a measure of sensitivity to PARP inhibitor treatment, such as mutations in DNA repair genes, their expression levels, as well as mutational and genomic signatures of HR deficiency. Replication stress markers and transcriptome profiles are complementary means of evaluating PARP inhibitor response given the importance of PARP1 for replication fork stability and gene expression regulation.

#### Homologous recombination (HR) deficiency

Germline or somatic mutations in DNA repair genes as well as their transcriptional down-regulation are frequently used as biomarkers of PARP inhibitor response. Ten percent to 15% of breast and ovarian cancer patients carry germline mutations in HR genes *BRCA1* and *BRCA2* (Gallagher et al. 2011; Mavaddat et al. 2012; Nik-Zainal et al. 2016). Strikingly, 75% of germline mutations in metastatic cancers affect DNA repair genes such as *MUTYH*, *BRCA2*, *CHEK2*, and *BRCA1* (Robinson et al. 2017). Moreover, *BRCA1/2*, *ATM*, and *CHEK2* are the most frequently mutated DNA repair genes in somatic cancer cells (Heeke et al. 2018). In addition to being directly inactivated by mutation, *BRCA1* and *RAD51C* were also found to be down-regulated through promoter hypermethylation in breast and ovarian cancer (Chiang et al. 2006; The Cancer Genome Atlas Research Network 2011; Lips et al. 2013; Timms et al. 2014; Polak et al. 2017; Bernards et al. 2018; Castroviejo-Bermejo et al. 2018; Kondrashova et al. 2018). *BRCA1* promoter methylation confers the same degree of sensitivity to PARP inhibitors as *BRCA1* mutations (Veeck et al. 2010). Furthermore, the expression level of *BRCA1* was shown to be reduced due to depletion of the CDK12 kinase, which sensitizes breast and ovarian cancer cells to PARP inhibition (Bajrami et al. 2014). CDK12 regulates transcription of HR genes by suppressing intronic polyadenylation (Dubbury et al. 2018). CDK12 is often mutated in ovarian and prostate cancer and CDK12 deficiency may thus prove useful as a biomarker of PARP inhibitor response (The Cancer Genome Atlas Research Network 2011; Bajrami et al. 2014; Wu et al. 2018). A recent CRISPR screen identified *TP53-induced glycolysis and apoptosis regulator* (TIGAR) as another modulator of expression of HR genes (Fang et al. 2019a). TIGAR is amplified in different cancer types and its down-regulation sensitizes cancer cells to PARP inhibitors through inhibition of the pentose phosphate pathway, increase in ROS and DNA damage, down-regulation of *BRCA1/2* and *RAD51*, and induction of cellular senescence (Fang et al. 2019a).

HR deficiency can also be scored based on different mutational and genomic signatures. A mutational signature of HR deficiency in *BRCA* mutated breast, ovarian, and pancreatic cancers are large indels (3- to 50-bp insertions and deletions) with overlapping microhomology at breakpoint junctions that result from NHEJ as the alternative pathway of DSB repair (Alexandrov et al. 2013). NHEJ joins two broken DNA ends, which may lead to small insertions or deletions (indels) (Chang et al. 2017). In addition to microhomology-mediated indels as the main signature of BRCA1/2 deficiency, base substitutions and rearrangements also reflect an abrogation of DSB repair pathways (Nik-Zainal et al. 2016). Mutational signatures identified from whole-genome sequencing of breast, ovarian, and pancreatic cancers were used to generate an HRDetect tool that can predict HR deficiencies (Davies et al. 2017). However, mutational signatures are not prognostic of PARP inhibitor sensitivity in the case of tumors with restored HR, which harbor mutational signatures but are resistant to PARP inhibitors due to restoration of the HR pathway (see “Mechanisms of Resistance to PARP Inhibitors”; Mateo et al. 2019).

Genomic signatures of HR deficiency comprise loss of heterozygosity (LOH), telomeric allelic imbalances (TAIs), and large-scale state transitions (LSTs). LOH results in irreversible loss of one of the parental alleles in regions >15 Mb (Abkevich et al. 2012). TAI refers to unequal contribution of maternal and paternal telomeric DNA sequences (Birkbak et al. 2012). LSTs are defined as chromosomal breaks between adjacent regions of at least 10 Mb (Popova et al. 2012). LOH, TAIs, and LSTs were shown to correlate well with mutations in HR genes *BRCA1/2* in breast and ovarian cancer (Abkevich et al. 2012; Birkbak et al. 2012; Popova et al. 2012). All these genomic signatures of HR deficiency have been combined in a “homologous recombination deficiency” (HRD) score as a measure of genomic instability (Timms et al. 2014; Telli et al. 2016). HRDetect and HRD scores are both used to predict PARP inhibitor sensitivity in clinical settings.

Examining transcriptional signatures (or RNA) of cancer cells rather than their mutational signatures (or DNA) emerged as another means of predicting PARP inhibitor sensitivity. Gene expression profile derived from *BRCA* mutated ovarian cancers, termed “the BRCAness profile,” was found to correlate with platinum and PARP inhibitor sensitivity and was efficient in predicting platinum sensitivity of non-*BRCA* mutated ovarian cancers (Konstantinopoulos et al. 2010). Furthermore, gene expression profiles from cell lines depleted in different HR proteins revealed an HRD transcriptome signature that can predict HR deficiency and PARP inhibitor sensitivity (Peng et al. 2014).

Last, the cytological signature of HR deficiency is given by the number of RAD51 foci. Reduced RAD51 foci formation indicates HR deficiency and correlates with PARP inhibitor sensitivity, as shown in *BRCA* mutated breast tumor samples 2 h after 5 Gy of IR (Naipal et al. 2014), ovarian cancer cell lines 8 h after 4 Gy of IR (Shah et al. 2014), and breast cancer patient-derived xenografts (PDXs) (Castroviejo-Bermejo et al. 2018; Cruz et al. 2018). Unlike RAD51, γH2AX foci are not a reliable predictor of sensitivity to PARP inhibition as γH2AX foci may correlate positively or negatively with HR deficiency and PARP inhibitor sensitivity (Fong et al. 2009; Mukhopadhyay et al. 2010; Dale Rein et al. 2015; Michelena et al. 2018).

#### Replication stress

High levels of replication stress and depletion of replication-associated genes may render cancer cells sensitive to PARP inhibitors even in the absence of HR deficiency. For example, loss of *TP53* and *RB1* coupled with amplification of *MYC* generate replication stress and sensitize small cell lung cancer cells (SCLC) to PARP inhibitors (George et al. 2015; Sen et al. 2018). Schlafen 11 (SLFN11) is a recently identified biomarker of PARP inhibitor response in SCLC. SLFN11 binds to RPA in response to replication stress and blocks replication fork progression by changing chromatin structure (Murai et al. 2018). *SLFN11* overexpression correlates with PARP inhibitor sensitivity, as shown in SCLC PDXs treated with olaparib or talazoparib, as well as in SCLC patients treated with veliparib in combination with temozolomide (Allison Stewart et al. 2017; Lok et al. 2017).

Overexpression of the cytidine deaminase APOBEC3, which causes an increase in abasic sites at replication forks, is frequently encountered in cancer and was shown to sensitize cells to PARP inhibitors (Burns et al. 2013; Roberts et al. 2013; Nikkilä et al. 2017). Mutations in genes required for Okazaki fragment processing such as *FEN1* also sensitize cells to PARP inhibition, but *FEN1* is rarely mutated in cancer (Murai et al. 2012). Depletion of replication-associated proteins such as cohesin (SMC1, SMC3, and RAD21), cohesin-associated factors (ESCO1 and ESCO2), core replication machinery (MCM2/3/6 helicases), and topoisomerases (TOP2B and TOP3A) increases sensitivity to PARP inhibition, as shown in colon and breast cancer cell lines (McLellan et al. 2012; Bajrami et al. 2014).

A recent CRISPR screen revealed synthetic lethality between mutations in *RNASEH2* and PARP inhibition due to increased levels of replication-dependent DNA damage (Zimmermann et al. 2018). RNase H2 deficiency results in impaired ribonucleotide excision repair and accumulation of ribonucleotides that are cleaved by TOP1 (Zimmermann et al. 2018). Cleavage of these ribonucleotides produces nicks, covalent TOP1–DNA adducts, and ssDNA gaps that can act as PARP-trapping lesions, thus contributing to PARP inhibitor efficacy (Zimmermann et al. 2018). *RNASEH2B* deletions are frequently found in chronic lymphocytic leukemia and metastatic prostate cancer, which renders them more sensitive to PARP inhibition (Zimmermann et al. 2018).

#### Transcriptome profiles, PARP1 expression levels, and PARP1 activity

Given the important roles of PARP1 in transcription regulation (Kraus and Hottiger 2013), gene expression profiles from ovarian and breast cancer cell lines with known sensitivity to olaparib and rucaparib were used to derive a transcriptional algorithm that can predict sensitivity to PARP inhibitors (McGrail et al. 2017). The expression levels of PARP1 itself may also determine PARP inhibitor response. PARP1 expression is increased in different cancer types, particularly at advanced stages (Ossovskaya et al. 2010; Domagala et al. 2011; Mascolo et al. 2012; Bi et al. 2013; Bieche et al. 2013; Gan et al. 2013; Salemi et al. 2013; Dziaman et al. 2014; Park et al. 2015; Zhai et al. 2015; Li et al. 2016; Hou et al. 2018), and in some cases correlates positively with the cytotoxic effects of PARP inhibition (Byers et al. 2012; Kukolj et al. 2017).

PARP1 catalytic activity is enhanced by the receptor tyrosine kinase c-Met-mediated phosphorylation on Y907, which in turn reduces PARP inhibitor binding. Blocking PARP1 phosphorylation with a c-Met inhibitor can increase the efficacy of PARP inhibitors (Du et al. 2016). Endogenous inhibition of PARP activity through increased levels of NADP^+^ was shown to render ovarian cancer cells hypersensitive to PARP inhibitors irrespective of the *BRCA* status, suggesting that NADP^+^ levels could also be used as a biomarker of PARP inhibitor sensitivity (Bian et al. 2019).

## Clinical studies with PARP inhibitors

The very first clinical trials demonstrated the efficacy of the PARP inhibitor olaparib in breast and ovarian cancer patients carrying germline mutations in *BRCA1/2*, thus supporting the rationale for synthetic lethality (Fong et al. 2009; Audeh et al. 2010; Tutt et al. 2010). The phase 1 trial showed the antitumor activity of olaparib at 400 mg twice daily and acceptable adverse effects (nausea, fatigue, vomiting, taste alteration, anorexia) (Table 1; Fong et al. 2009). The PARP inhibitor niraparib showed antitumor activity at 300 mg daily with more pronounced hematologic adverse effects compared with olaparib (anemia, thrombocytopenia, and neutropenia) (Sandhu et al. 2013). Rucaparib showed partial or complete response in ovarian, breast, and pancreatic cancer patients given 600 mg twice daily, with fatigue, nausea, anemia, and vomiting as the most common adverse effects (Kristeleit et al. 2017). Talazoparib administered at the recommended dose of 1 mg/d demonstrated high antitumor activity in *BRCA* mutated breast and ovarian cancer patients with fatigue, anemia, and thrombocytopenia as the most pronounced adverse effects (de Bono et al. 2017). Clinically recommended doses and the severity of side effects correlate with the PARP inhibitor trapping potency; talazoparib as the strongest PARP trapper has the lowest recommended dose and shows the highest occurrence of anemia (de Bono et al. 2017; Litton et al. 2018; Pilié et al. 2019a).

**Table 1.**
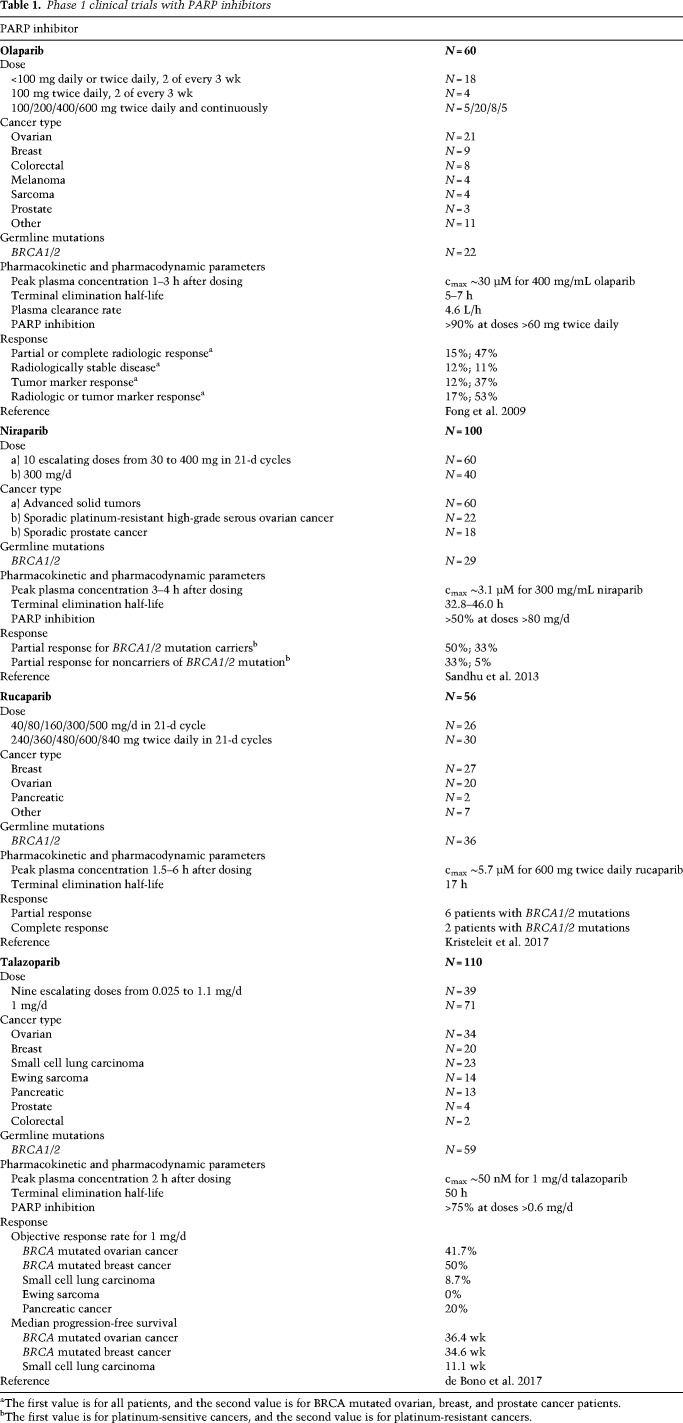
Phase 1 clinical trials with PARP inhibitors

Olaparib treatment in advanced breast or ovarian cancer patients with *BRCA1/2* germline mutations showed a 41% and a 33% objective response rate defined as the proportion of patients with tumor size reduction of a predefined amount and for a minimum time period (Table 2; Audeh et al. 2010; Tutt et al. 2010). Olaparib administered in high-grade serous and/or undifferentiated ovarian cancer patients showed a 41% and 24% objective response rate with or without *BRCA1/2* mutations; however, there was no response in TNBC patients (Gelmon et al. 2011). A study comparing olaparib with placebo in platinum-sensitive, relapsed, high-grade serous ovarian cancer patients who had received two or more platinum-based regimens showed longer median progression-free survival from 4.3 to 11.2 mo for *BRCA* mutated cancer and from 5.5 to 7.4 mo for wild-type (WT) *BRCA* (Ledermann et al. 2012, 2014). However, the overall survival of olaparib-treated versus placebo patients was not significantly different after 5 yr (Ledermann et al. 2016). Long-term responders to olaparib with progression-free survival >2 yr had a prevalence of *BRCA2* mutations and a high HRD score, confirming that mutations in HR genes such as *BRCA2* and HRD score can be used as predictive biomarkers for PARP inhibitor response (Lheureux et al. 2017b). A study with platinum-resistant ovarian and breast cancer patients with three or more chemotherapy regimens for metastatic disease, all carrying *BRCA1/2* mutations, showed a 31.1% and 12.9% tumor response rate to olaparib, indicating that olaparib may indeed be more effective in ovarian than breast cancer (Kaufman et al. 2015). Based on these studies, olaparib was approved in 2014 by EMA for the treatment of germline *BRCA* mutated ovarian cancer after three or more lines of chemotherapy, and by the FDA for the maintenance treatment of *BRCA* mutated ovarian cancer patients who have responded to platinum-based chemotherapy. The currently approved dose is 300 mg twice daily.

**Table 2.**
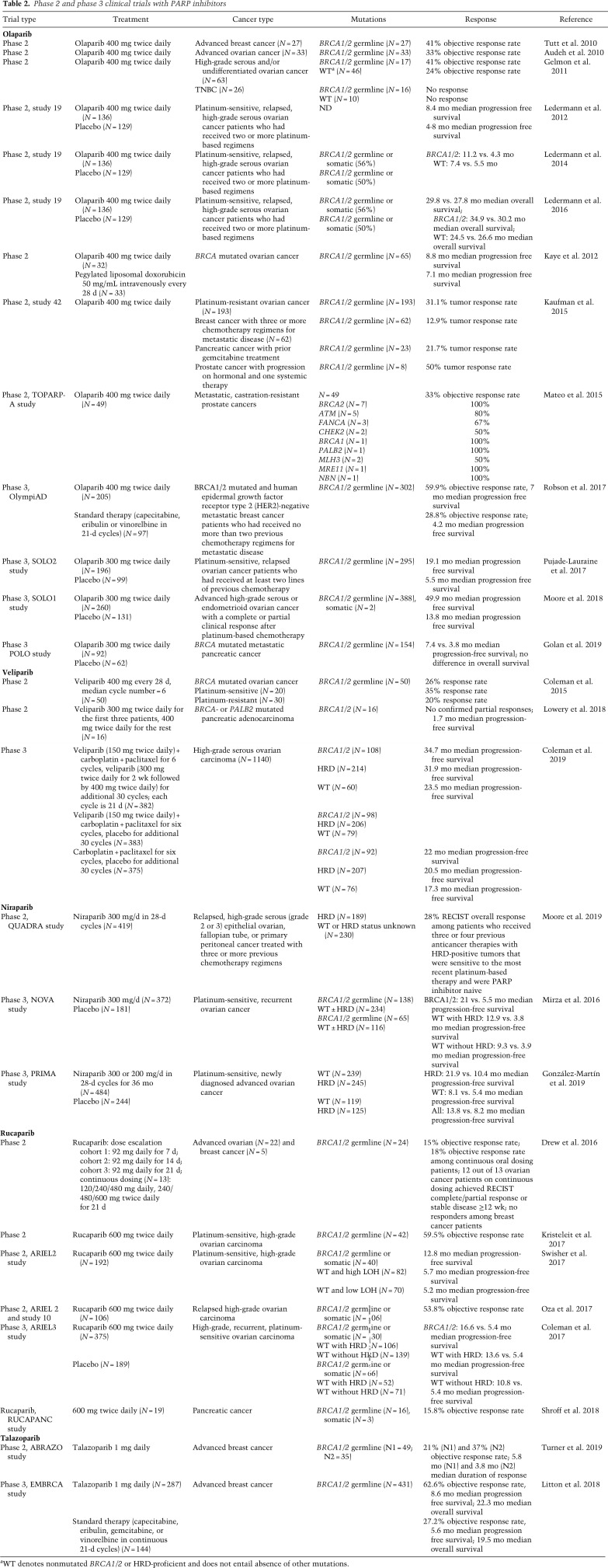
Phase 2 and phase 3 clinical trials with PARP inhibitors

In 2017, olaparib was approved by the FDA as maintenance therapy in platinum-sensitive high-grade ovarian cancer patients irrespective of the *BRCA* status, based on phase 3 clinical trials that showed longer median progression-free survival compared with placebo, from 5.5 mo to 19.1 mo (Pujade-Lauraine et al. 2017) or from 13.8 to 49.9 mo in patients with germline BRCA1/2 mutations (Table 2; Moore et al. 2018). In a phase-3 trial focusing on *BRCA* mutated and HER2-negative metastatic breast cancer patients, olaparib was compared with standard single-agent therapy (e.g., microtubule inhibitors, nucleoside, or fluoropyrimidine analogs) and showed a longer median progression-free survival and 59.9% objective response rate compared with 28.8% for patients receiving standard therapy (Robson et al. 2017). As a result, olaparib was approved by the FDA for germline *BRCA* mutated metastatic breast cancer in 2018. It remains to be clarified whether olaparib also confers an advantage over standard chemotherapy in ovarian cancer, given that one clinical trial in advanced ovarian cancer patients with *BRCA1/2* mutations whose disease had progressed or recurred after the use of platinum-based chemotherapy showed that the effect of olaparib was not superior to pegylated liposomal doxorubicin (PLD) (Kaye et al. 2012). Nevertheless, chemotherapy is invariably associated with side effects on normal cells, which makes PARP inhibitors the therapy of choice even without appreciable differences in overall survival.

Rucaparib administered at 600 mg twice daily in platinum-sensitive, high-grade recurrent ovarian carcinoma patients showed longer median progression-free survival in *BRCA* mutated cancer compared with WT (12.8 mo vs. ∼5 mo) and a 53.8% objective response rate in *BRCA* mutated patients (Table 2; Oza et al. 2017; Swisher et al. 2017). As a result, rucaparib was approved in 2016 for advanced ovarian cancer with germline and somatic *BRCA1/2* mutations. Phase 3 trials showed that rucaparib (600 mg twice daily) and niraparib (300 mg once daily) increased the median duration of progression-free survival compared with placebo, from ∼5 mo for placebo to 16.6 or 21 mo for rucaparib and niraparib (Mirza et al. 2016; Coleman et al. 2017). Niraparib was effective not only in the treatment of recurrent ovarian cancer but also newly diagnosed advanced ovarian cancer (Mirza et al. 2016; González-Martin et al. 2019). While the effect was more pronounced in *BRCA* mutated cancers, the patients without *BRCA* mutations also showed a good response to niraparib and rucaparib, indicating that PARP inhibitors can be effective irrespective of the *BRCA* status (Mirza et al. 2016; Coleman et al. 2017; González-Martin et al. 2019). Niraparib and rucaparib were approved in 2017 and 2018 by the FDA and the EMA as a maintenance treatment of recurrent, epithelial ovarian, fallopian tube, or primary peritoneal cancer irrespective of the *BRCA* status.

Talazoparib was approved in 2018 for *BRCA* mutated and HER2-negative breast cancer. Talazoparib administered at 1 mg daily showed a 21%–37% objective response rate in *BRCA* mutated advanced breast cancer patients (Table 2; Turner et al. 2019). Compared with standard single-agent therapy in *BRCA* mutated advanced breast cancer patients, talazoparib showed longer median progression-free survival and a 62.6% objective response rate compared with 27.2% for patients receiving standard therapy (Litton et al. 2018).

Veliparib applied as monotherapy did not improve clinical outcomes in ovarian and pancreatic cancer patients (Coleman et al. 2015; Lowery et al. 2018) or in combination with carboplatin/paclitaxel in TNBC (Table 2; Loibl et al. 2018). However, the most recent phase 3 study in high-grade serous ovarian carcinoma showed increased progression-free survival for veliparib in combination with carboplatin and paclitaxel followed by veliparib maintenance therapy compared with carboplatin and paclitaxel alone (Coleman et al. 2019). Veliparib is so far the only clinically relevant PARP inhibitor that is tolerated in combination with standard doses of chemotherapy.

PARP inhibitors have also shown good response in prostate and pancreatic patients. Germline *BRCA2* mutations in prostate cancer patients are associated with worse clinical outcomes (Castro et al. 2013; Taylor et al. 2019). Olaparib administered in *BRCA* mutated pancreatic and prostate cancer patients elicited a 21.7% and 50% tumor response rate (Kaufman et al. 2015). Another study showed a 33% response rate to olaparib in prostate cancer patients, where most of the responders had *BRCA2* or *ATM* mutations (Mateo et al. 2015). A phase 3 trial in metastatic pancreatic cancer patients showed longer median progression-free survival for olaparib versus placebo (7.4 vs. 3.8 mo) (Golan et al. 2019). Rucaparib showed a 15.8% response rate in *BRCA* mutated pancreatic cancer patients (Shroff et al. 2018). To date, PARP inhibitors have not yielded promising results in the treatment of metastatic gastric cancer, according to a phase 3 study where olaparib in combination with paclitaxel (taxane drug) did not improve overall survival (Bang et al. 2017). However, PARP inhibitors have shown encouraging results in small cell lung cancer (SCLC). Veliparib combined with temozolomide showed improved objective response rate in SCLC patients (Pietanza et al. 2018), while talazoparib alone or in combination with temozolomide yielded promising results in PDX models of SCLC (Lok et al. 2017; Laird et al. 2018).

PARG inhibitors have not yet reached clinical trials due to low metabolic stability. However, newly identified PARG inhibitors COH34 and JA2131 exhibit favorable pharmacokinetic properties and may enter clinical studies (Chen and Yu 2019; Houl et al. 2019).

## Mechanisms of resistance to PARP inhibitors

### Restoration of the HR pathway

Resistance to chemotherapy is a frequent problem in clinical practice that also affects PARP inhibitors (Gogola et al. 2019; Noordermeer and van Attikum 2019). The most common avenue of PARP inhibitor resistance is restoration of the HR pathway. The first example of PARP inhibitor resistance was identified in Capan-1 cells derived from a pancreatic epithelial tumour, which had an intragenic deletion in *BRCA2* of 458 bp resulting in removal of the inactivating frameshift mutation and expression of an almost full-length BRCA2 protein lacking 153 amino acids (Edwards et al. 2008). Since then, many examples of secondary mutations in *BRCA1/2*, as well as *RAD51C*, *RAD51D*, and *PALB2*, that genetically revert the mutation and restore functional full-length protein have been reported in breast, ovarian, pancreatic, and prostate carcinoma (Sakai et al. 2008; Norquist et al. 2011; Barber et al. 2013; Patch et al. 2015; Christie et al. 2017; Goodall et al. 2017; Kondrashova et al. 2017; Lheureux et al. 2017a; Pishvaian et al. 2017; Quigley et al. 2017; Weigelt et al. 2017; Lin et al. 2019). Platinum and cisplatin chemotherapy seem to select for these secondary mutation events (Sakai et al. 2008; Norquist et al. 2011).

Secondary mutations in *BRCA1* may result in the expression of functional hypomorphic variants in PARP inhibitor-resistant patients (Drost et al. 2016; Wang et al. 2016c). Such mutant BRCA1 protein may be stabilized by the heat shock chaperone HSP90 as shown in MDA-MB-436 breast cancer cells (Johnson et al. 2013).

Increased levels of RAD51 as the core component of the HR pathway were also shown to contribute to PARP inhibitor resistance in TNBC (Liu et al. 2017). In TNBC cell lines, down-regulation of the early mitotic inhibitor 1 (EMI1), which assembles a ubiquitin ligase complex to degrade RAD51, induces PARP inhibitor resistance (Marzio et al. 2019).

Finally, inactivation of different NHEJ-promoting factors that inhibit DNA end resection, such as 53BP1, RIF1, PTIP, Artemis, REV7 (MAD2L2), the Shieldin complex (SHLD1–3 and REV7), and the CTC1–STN1–TEN1 (CST) complex, can also lead to a partial restoration of HR in BRCA1-deficient cells and mammary tumors (Bunting et al. 2010; Callen et al. 2013; Chapman et al. 2013; Escribano-Diaz et al. 2013; Jaspers et al. 2013; Zimmermann et al. 2013; Wang et al. 2014; Xu et al. 2015; Barazas et al. 2018; Dev et al. 2018; Ghezraoui et al. 2018; Gupta et al. 2018; Noordermeer et al. 2018). Furthermore, inactivation of dynein DYNLL1 and the HELB helicase can also promote end resection and restore the HR pathway independent of 53BP1, resulting in PARP inhibitor resistance in BRCA1-deficient cells (Tkáč et al. 2016; Dev et al. 2018; He et al. 2018). Of note, while *BRCA1* mutations are compatible with HR restoration upon inactivation of NHEJ-promoting factors, restoration of the HR pathway fully depends on functional BRCA2 (Bouwman et al. 2010).

### Stabilization of replication forks

In BRCA1/2-deficient cells, stabilization of replication forks independent of HR restoration and DSB repair emerged as an alternative mechanism of PARP inhibitor resistance (Ray Chaudhuri et al. 2016). PARP1, BRCA1, and BRCA2 protect stalled forks from nucleolytic degradation and enable their restart after DNA damage; their loss therefore leads to fork degradation (Lomonosov et al. 2003; Bryant et al. 2009; Ray Chaudhuri et al. 2012; Schlacher et al. 2012; Ying et al. 2012; Berti et al. 2013; Pathania et al. 2014; Ronson et al. 2018). Compensatory mechanisms of restoring fork protection in the absence of BRCA1/2 or PARP1 may be at play in PARP inhibitor-resistant cancers. For example, loss or inhibition of PTIP, a downstream effector of 53BP1, protects forks from degradation in BRCA2-deficient cells by inhibiting the recruitment of the MRE11 nuclease to stalled forks, resulting in PARP inhibitor resistance (Ray Chaudhuri et al. 2016). Furthermore, reduced expression of the Polycomb protein EZH2 in *BRCA2* mutated cancer results in fork stabilization due to reduced H3K27 methylation and reduced recruitment of the MUS81 nuclease as a result (Rondinelli et al. 2017). Depletion of fork remodeling factors such as SMARCAL1, ZRANB3, and HLTF, which promote MRE11-dependent fork degradation, also reduces sensitivity to olaparib (Taglialatela et al. 2017). Increased expression of miR-493-5p was shown to induce resistance to PARP inhibitors in *BRCA2* mutated carcinomas by down-regulating MRE11 and EXO1 and thereby stabilizing replication forks (Meghani et al. 2018). While *SLFN11* overexpression is used as a biomarker of PARP inhibitor sensitivity (Allison Stewart et al. 2017; Lok et al. 2017), *SLFN11* is inactivated in 50% of cancers and frequently silenced due to promoter methylation (Nogales et al. 2016; Murai et al. 2018). SLFN11 repression occurs frequently in talazoparib-resistant cancer cell lines and was linked with impaired S-phase arrest and G2 progression, which may also be due to fork stabilization (Murai et al. 2016, 2018).

### PARP1 and PARG mutations

PARP1 mutations within and outside the DNA-binding domain were found to confer resistance to PARP inhibitors by hindering PARP trapping (Pettitt et al. 2013, 2018). A clinically relevant PARP mutation found in an olaparib-resistant ovarian cancer patient occurs within the WGR domain (R591C) (Pettitt et al. 2018). The mutated residue is critical for interdomain communication between the WGR and the DNA-binding domain; while being proficient in DNA binding, the PARP1 R591C mutant dissociates rapidly from DNA damage sites, indicating inefficient PARP DNA trapping (Pettitt et al. 2018). PARP1 mutations can only confer PARP inhibitor resistance in HR-proficient cells or *BRCA1* mutated cells that have residual BRCA1 activity, as *PARP1* and *BRCA1* mutations are synthetic lethal (Pettitt et al. 2018).

PARP1 depletion was shown to reduce sensitivity to PARP inhibitors in DT40 cells (Murai et al. 2012). *BRCA1* mutated breast cancer PDX models resistant to PARP inhibitors showed reduced PARP1 expression levels (Dev et al. 2018). However, PARP inhibitor resistance due to reduced PARP1 expression levels might not be very common in vivo due to the high cellular abundance of PARP1 (Thomas et al. 2018).

PARG mutations were identified in TNBC and high-grade serous ovarian cancers eligible for PARP inhibitor treatment (Gogola et al. 2018). On the one hand, PARG depletion was shown to reduce sensitivity of HeLa cells to PARP inhibitors combined with DNA-damaging agents or mouse *Brca2* mutated cells to PARP inhibitors alone (Feng and Koh 2013; Gogola et al. 2018). PARG deficiency may contribute to PARP inhibitor resistance by increasing PARP1 auto-PARylation that allows PARP1 dissociation from DNA (i.e., reduction in PARP trapping) and restoration of PARP signaling (Gogola et al. 2018). On the other hand, down-regulation of PARG due to loss of the RNA stabilizing protein HuR (ELAV-L1) was shown to enhance sensitivity to PARP inhibitors in pancreatic ductal carcinoma (Chand et al. 2017). Increased PARylation may sensitize cells to PARP inhibitors by enhancing PARP1 trapping and promoting DNA damage accumulation (Chand et al. 2017). The variable effects of PARG depletion on reduction or increase in PARP trapping may be due to the nature of stabilized PAR polymers or the site of their attachment.

### Drug efflux

Overexpression of ATP-binding cassette (ABC) drug transporters is often associated with drug resistance (Patch et al. 2015). Olaparib and rucaparib, but not veliparib, are substrates of the ATP-dependent drug efflux P-glycoprotein (P-gp) pump (also known as MDR1, encoded by *ABCB1*) (Lawlor et al. 2014; Henneman et al. 2015; Parrish et al. 2015). Long-term treatment with PARP inhibitors leads to the up-regulation of P-gp, thereby reducing the intracellular concentrations of PARP inhibitors (Rottenberg et al. 2008). Coadministration of the pump inhibitor verapamil, elacridar, or tariquidar can resensitize cancer cells to PARP inhibitors (Rottenberg et al. 2008; Vaidyanathan et al. 2016).

## PARP inhibitor combination therapies

Frequently acquired resistance to PARP inhibitors has spurred efforts to combine PARP inhibitors with other agents (Dréan et al. 2016; Pilié et al. 2019b). Phase 1 clinical trials with PARP inhibitors in combination with chemotherapeutic agents such as temozolomide, cisplatin, carboplatin, paclitaxel, gemcitabine, or the topoisomerase inhibitor I topotecan showed severe myelosuppression in the form of neutropenia and thrombocytopenia as a side effect (Matulonis and Monk 2017; Yap et al. 2019). Veliparib is the only clinically relevant PARP inhibitor that can be combined with chemotherapy (see “Clinical Studies with PARP Inhibitors”). Administration of PARP inhibitors following chemotherapy has proven to be a better strategy (Ledermann et al. 2012; Coleman et al. 2015, 2017; Mirza et al. 2016; Oza et al. 2017; Pujade-Lauraine et al. 2017; Robson et al. 2017; Swisher et al. 2017; Moore et al. 2018). Moreover, mutations that cause resistance to PARP inhibitors, such as loss of NHEJ factors, PARP1, or PARG, were shown to sensitize cells to different DNA-damaging agents such as radiotherapy, topoisomerase I inhibitors and temozolomide (Murai et al. 2014b; Gogola et al. 2018; Barazas et al. 2019).

### Cell cycle checkpoint inhibitors

Combination of PARP inhibitors with cell cycle checkpoint inhibitors is a promising strategy in overcoming PARP inhibitor resistance caused by replication fork stabilization (Kim et al. 2017; Yazinski et al. 2017; Haynes et al. 2018). The S/G2 checkpoint is essential to allow repair of DSBs induced by PARP inhibitors and to prevent premature mitotic entry and mitotic catastrophe. Inhibition of the S/G2 checkpoint kinases ATR and CHK1 results in unscheduled replication origin firing, exhausts nuclear RPA pools due to excess ssDNA, depletes dNTPs, and allows mitotic entry in the presence of underreplicated DNA and unrepaired DNA damage from interphase (Toledo et al. 2017; Lecona and Fernandez-Capetillo 2018). The combination of PARP inhibitors and ATR or CHK1 inhibitors in HR-deficient cells causes the release of the G2/M arrested cells, accumulation of chromosomal breaks, and aberrations in mitosis followed by cell death (Kim et al. 2017; Fang et al. 2019b). Chromosomal breaks in mitosis, indicative of unrepaired DSBs, are more pronounced for the PARP–ATR inhibitor combination, which is also more effective in inducing tumor regression in ovarian cancer PDX models (Kim et al. 2017). The ATR inhibitor overcomes fork stabilization in BRCA-deficient cells by preventing RAD51 loading onto stalled forks and triggering MRE11-mediated fork degradation, as shown in PARP-resistant ovarian cancer cells and PDXs (Yazinski et al. 2017). The ATR inhibitor can also overcome resistance to talazoparib due to *SLFN11* repression (Murai et al. 2016). Although PARG inhibitors have not yet reached clinical trials, they have shown synergistic effects with CHK1 inhibitors in ovarian cancer cell lines and ovarian PDX models (Pillay et al. 2019).

Furthermore, PARP inhibitors were successfully combined with WEE1 kinase inhibitors (Lallo et al. 2018; Parsels et al. 2018; Fang et al. 2019b). WEE1 kinase regulates S phase and G2/M progression by inhibiting cell cycle-dependent kinases CDK1 and CDK2. The combination of PARP and WEE1 inhibitors abrogates G2 arrest and induces mitotic catastrophe, yielding promising results in small cell lung cancer (Lallo et al. 2018), *KRAS* mutated nonsmall cell lung cancers (Parsels et al. 2018), gastric cancer (Lin et al. 2018), and *TP53* mutated cancer (Meng et al. 2018). Sequential rather than concurrent inhibition of PARP and ATR or WEE1 is preferable in a clinical setting due to lower toxicity while preserving efficacy (Fang et al. 2019b).

PARP inhibitors also synergize with the pan-CDK inhibitor dinaciclib in reducing *MYC* expression in TNBC and other cancer types, resulting in down-regulation of HR genes and induction of DNA damage (Carey et al. 2018).

### Inhibitors of transcription regulators and epigenetic modifiers

Inhibition of BRD4 as the global transcription regulator is synthetic lethal with PARP inhibitors (Karakashev et al. 2017; Yang et al. 2017; Sun et al. 2018). BRD4 inhibition induces HR deficiency independent of the *BRCA* status by impairing *CTIP, BRCA1*, and *RAD51* expression, inducing DNA damage and eventually resulting in mitotic catastrophe in various cancer cell lines and PDX models (Karakashev et al. 2017; Yang et al. 2017; Sun et al. 2018). Inhibition of the Polycomb protein EZH2 synergizes with PARP inhibitors in *BRCA* mutated breast and ovarian cancer cells (Yamaguchi et al. 2018). Histone deacetylase (HDAC) inhibitors synergize with PARP inhibitors through the down-regulation of HR genes and an increase in DNA damage in PTEN-positive TNBC and prostate cancer (Chao and Goodman 2014; Min et al. 2015). EZH2 and HDAC inhibitors may restore PARP inhibitor sensitivity by restoring SLFN11 expression in SCLC (Gardner et al. 2017; Tang et al. 2018). The combination with inhibitors of DNA methyltransferase 1 (DNMT1) was shown to enhance the PARP inhibitor effect in TNBC through an increase in DSBs due to PARP retention at DNA damage sites (Muvarak et al. 2016).

### Agents that pharmacologically induce HR deficiency: androgen receptor inhibitors, PI3K–AKT–mTOR inhibitors, and antiangiogenic agents

PARP inhibitors have been successfully combined with agents that pharmacologically recreate HR deficiency and BRCAness. For example, the combination of olaparib with the androgen receptor (AR) inhibitors enzalutamide or bicalutamid is effective in HR-proficient prostate cancer due to suppression of HR gene expression (Asim et al. 2017; Li et al. 2017). Olaparib in combination with the AR antagonist abiraterone in metastatic prostate cancer patients extended median progression-free survival from 8.2 mo (abiraterone alone) to 13.8 mo regardless of the HR mutation status (Table 3; Clarke et al. 2018).

**Table 3.**
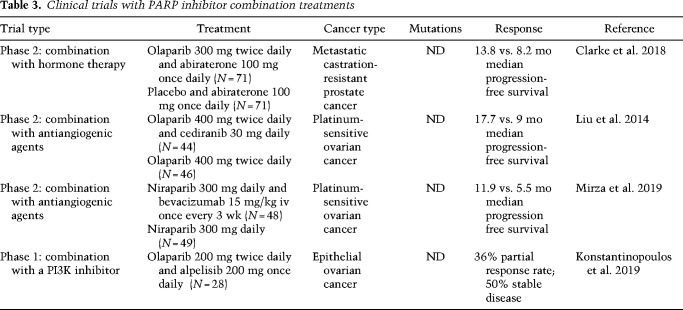
Clinical trials with PARP inhibitor combination treatments

PARP inhibitors further synergize with inhibitors of the PI3K pathway, as shown in BRCA-proficient TNBC, *BRCA1* mutated breast cancer mouse models, *PIK3CA* mutated ovarian cancer cells, and *PTEN* mutated endometrial cancer cells (Ibrahim et al. 2012; Juvekar et al. 2012; Wang et al. 2016b; Philip et al. 2017). A phase I clinical trial revealed synergistic effects between olaparib and the PI3K inhibitor alpelisib in epithelial ovarian cancer, which are most likely based on HR suppression through reduced RAD51 protein levels and RAD51 foci formation (Konstantinopoulos et al. 2019). The combination of talazoparib and the mTOR inhibitor everolimus synergistically kills BRCA-proficient TNBC by inducing HR deficiency through repression of the histone methyltransferase SUV39H1 (Mo et al. 2016).

Combination with antiangiogenic agents may sensitize tumors by inducing hypoxia and thereby reducing the expression of HR genes (Bindra et al. 2005). Hypoxia induces reduction in binding of the activating transcription factor E2F1 to the *BRCA1* promoter, resulting in reduced *BRCA1* expression (Bindra et al. 2005). PARP inhibition also reduces E2F1 genomic binding and expression of the E2F1 target genes (Byers et al. 2012; Schiewer et al. 2018) and may thereby synergize with hypoxic effects induced by antiangiogenic agents. A successful example of such a synergy is given by the combination of olaparib with the pan-VEGF inhibitor cediranib, which increased median progression-free survival from 9 to 17.7 mo compared with olaparib alone in platinum-sensitive ovarian cancer (Liu et al. 2014). Another VEGF inhibitor bevacizumab has also shown promising results in combination with niraparib in platinum-sensitive ovarian cancer patients (Mirza et al. 2019).

### Immune checkpoint inhibitors

Replication stress induced by PARP inhibitors was recently shown to stimulate the expression of type I interferons by activating the cGAS–STING pathway (Shen et al. 2019). DNA damage or DNA repair defects caused by *BRCA* or *ERCC1* mutations can also activate this pathway and thereby potentiate PARP inhibitor effects in ERCC1-deficient nonsmall cell lung cancer, BRCA1-deficient TNBC, and ovarian cancer (Ding et al. 2018; Chabanon et al. 2019). The cGAS–STING pathway is activated by cytosolic DNA fragments coming from micronuclei or destabilized replication forks bearing ssDNA and unresolved DNA lesions (Harding et al. 2017; Mackenzie et al. 2017; Parkes et al. 2017). Nucleases such as MRE11, EXO1, and CtIP are required for production of DNA fragments and activation of the cGAS–STING pathway (Shen et al. 2019). Stimulation of interferon production by the cGAS–STING pathway promotes antitumor immunity in immunogenic tumors but also triggers the expression of immune checkpoints such as PD-L1 (Deng et al. 2014; Parkes et al. 2017). PD-L1 is a ligand that binds to the PD-1 receptor on T cells and suppresses T-cell proliferation and cytokine release. Immune checkpoint inhibitors are antibodies that block PD-L1 to restore T-cell function. Immunotherapy with immune checkpoint inhibitors relies on the activation of the cGAS–STING pathway (Harding et al. 2017). Therefore, combining PARP inhibitors that activate the cGAS–STING pathway with immune checkpoint inhibitors has proven effective as an antitumor therapy. PARP inhibitors combined with antibodies against PD-L1 show synergistic effects in breast cancer cells and PDXs (Jiao et al. 2017) as well as ovarian and colon-cancer mouse models (Ding et al. 2018; Shen et al. 2019). Phase II trials of the PD-L1 antibody durvalumab or pembrolizumab in combination with olaparib or niraparib showed clinical response in patients with advanced SCLC, germline *BRCA1/2* mutated breast and platinum-sensitive ovarian cancer, but also in prostate cancer and platinum-resistant ovarian cancer patients irrespective of *BRCA* mutations (Pilié et al. 2019a).

## Concluding remarks

PARP and PARG are critical regulators of DNA damage response and replication fork stability. Cancer cells experience high levels of replication stress and often harbor germline or somatic mutations in DNA damage response genes. Replication stress compromises replication fork stability, whereas mutations in homologous recombination genes prevent the restoration of fork stability. PARP and PARG inhibitors exploit and exacerbate these vulnerabilities of cancer cells by destabilizing replication forks and inducing DNA damage. The original concept of synthetic lethality between PARP inhibitors and breast and ovarian cancer mutations in *BRCA1/2* has now expanded to include a range of cancer types exhibiting homologous recombination deficiency and replication stress. PARP inhibitors have demonstrated the success of the “DNA damage response synthetic lethality” paradigm in various clinical trials by markedly extending disease progression-free survival while being well-tolerated. As clinical trials exploring further indications and combination treatments with PARP inhibitors are ongoing and PARG inhibitor trials still pending, the PARP community should invest further efforts into understanding on a molecular and cellular level how PARP and PARG maintain replication fork integrity and how replication stress and genomic instability resulting from their inhibition instigate mitotic defects and cell death by replication and mitotic catastrophe. This would advance new biomarkers of PARP inhibitor sensitivity and would guide new combination strategies.
